# The next generation of spatial transcriptomics and AI to understand complex interactions between glial cells

**DOI:** 10.1002/alz70855_107838

**Published:** 2025-12-24

**Authors:** Sudeshna Das

**Affiliations:** ^1^ Massachusetts General Hospital, Boston, MA, USA

## Abstract

Emerging research highlights the significant contributions of non‐neuronal cells—astrocytes, microglia, and oligodendrocytes—in Alzheimer's disease (AD). These glial cells are not only implicated in neuroinflammation, synaptic dysfunction, and neurodegeneration but also engage in highly dynamic crosstalk that modulates key pathological features such as amyloid‐beta (Aβ) deposition, tau pathology, and demyelination. While astrocyte‐microglia signaling in AD has been extensively studied, less is known about their interactions with neurons and oligodendrocytes. Furthermore, mechanistic pathways involving simultaneous interactions among astrocytes, microglia, and oligodendrocytes—in contexts such as inflammatory signaling, metabolic coupling, or Aβ/tau propagation—are sparsely investigated. Understanding this crosstalk is crucial to unraveling the complex cellular interplay that drives AD progression and identifying novel therapeutic targets.

For this session, we will invite experts on astrocytes, microglia, and oligodendrocytes biology to provide the latest developments on understanding the neuroimmune interactions mediating intercellular communication in AD pathology. To comment on the role of microglia, we propose to invite Dr. Beth Stevens from the Broad Institute of MIT, whose research focuses on understanding how glia interact with synapses. To comment on astrocytes, we will invite Dr. Eduardo Zimmer from Universidade Federal in Brazil, who studies crosstalk between microglia and astrocytes. Dr. Shin Kang from the Temple University will discuss the contribution of an oligodendroglia‐derived cytokine to AD pathology, which involves cell‐cell interactions between oligodendroglia and microglia. Finally, Dr. Das, who is a computational biologist, will discuss how the next generation of spatial transcriptomics and AI can be deployed to understand the complex interactions.

